# Mitochondrial ancestry of medieval individuals carelessly interred in a multiple burial from southeastern Romania

**DOI:** 10.1038/s41598-018-37760-8

**Published:** 2019-01-30

**Authors:** Ioana Rusu, Alessandra Modi, Claudia Radu, Cristina Mircea, Adriana Vulpoi, Cătălin Dobrinescu, Vitalie Bodolică, Tiberiu Potârniche, Octavian Popescu, David Caramelli, Beatrice Kelemen

**Affiliations:** 1 Molecular Biology Center, Interdisciplinary Research Institute on Bio-Nano-Sciences, Babeș-Bolyai University, 400271 Cluj Napoca, Romania; 2 Department of Molecular Biology and Biotechnology, Faculty of Biology and Geology, Babeș-Bolyai University, 400006 Cluj Napoca, Romania; 3 Dipartimento di Biologia, Università di Firenze, 50122 Florence, Italy; 4 Department of Ancient History and Archaeology, Faculty of History and Philosophy, Babeș-Bolyai University, 400084 Cluj Napoca, Romania; 5 Nanostructured Materials and Bio-Nano-Interfaces Center, Interdisciplinary Research Institute on Bio-Nano-Sciences, Babeș-Bolyai University, 400271 Cluj Napoca, Romania; 6 Department of Research-Development and Projects, Museum of National History and Archeology, 900745 Constanța, Romania; 7 Institute of Biology Bucharest, Romanian Academy, 060031 Bucharest, Romania

## Abstract

The historical province of Dobruja, located in southeastern Romania, has experienced intense human population movement, invasions, and conflictual episodes during the Middle Ages, being an important intersection point between Asia and Europe. The most informative source of maternal population histories is the complete mitochondrial genome of archaeological specimens, but currently, there is insufficient ancient DNA data available for the medieval period in this geographical region to complement the archaeological findings. In this study, we reconstructed, by using Next Generation Sequencing, the entire mitochondrial genomes (mitogenomes) of six medieval individuals neglectfully buried in a multiple burial from Capidava necropolis (Dobruja), some presenting signs of a violent death. Six distinct maternal lineages (H11a1, U4d2, J1c15, U6a1a1, T2b, and N1a3a) with different phylogenetic background were identified, pointing out the heterogeneous genetic aspect of the analyzed medieval group. Using population genetic analysis based on high-resolution mitochondrial data, we inferred the genetic affinities of the available medieval dataset from Capidava to other ancient Eurasian populations. The genetic data were integrated with the archaeological and anthropological information in order to sketch a small, local piece of the mosaic that is the image of medieval European population history.

## Introduction

Over thousands of years, the Lower Danube Basin witnessed striking social and cultural changes and several waves of human population migrations that have contributed to the shaping of the current European genetic landscape. Large-scale studies^1–4^ on the ancient DNA (aDNA) preserved in human archaeological remains, a direct proof of mankind’s historical past, have brought to light significant genetic evidence to answer some puzzling questions regarding complex past interactions. Most of them shed light on the genomic history of different European regions from the Mesolithic to the Bronze Age, whereas the genetic heritage mirrored in more recent time transects remains partly obscured due to the limited number of analyzed samples and/or populations. This scenario reflects the situation of southeastern Europe. Based on the genomes of four prehistoric samples from Romania, González Fortes *et al*.^5^ revealed that the Neolithization process in this area relied on the movement of both people and ideas. The analysis and interpretation of genome-wide aDNA data from more than 200 individuals inhabiting southeastern Europe between 12,000 and 500 BC, showed that this geographical region “served as a genetic contact zone between east and west over thousands of years^6^”. This pattern was also observed in a small medieval group of individuals from southeastern Romania (Dobruja)^7^. The genetic connections to Central Asia were reflected by the presence of the N9a haplotype in 2 out of 10 successfully analyzed samples^7^. Evidence from present-day maternal haplogroup diversity in the historical provinces of Romania showed that the Asian influence in Dobruja is greater than in the other regions, and suggested that the Carpathian Arch might have had an influential role in migration patterns^8^. Other findings from ancient mitochondrial DNA (mtDNA) research revealed the contribution of Hungarian Conquerors to the gene pool of the Carpathian Basin and suggested that most probably they descended from steppe nomadic people^9^. However, the archaeogenetic picture of medieval southeastern Europe is far from being complete.

The aim of the current study is to catch a glimpse into the maternal population history of the Lower Danube Basin by integrating full-length mitochondrial data with archaeological and anthropological evidence of a small group of medieval individuals (C58 archaeological complex) discovered in southeastern Romania (Capidava necropolis). This study is an attempt in understanding why these individuals were collectively buried in a particular fashion, which contrasts with the typical Christian medieval funerary practices observed at the same site, to evaluate the levels of variability within this group, and the relationships to other ancient populations from Eurasia.

Capidava fortress (Capidava village, Constanța County), a civil and military center after the Roman conquest, stands on the right bank of the Danube, halfway between Hârșova (Carsium fortress) and Cernavodă (Axiopolis fortress), the road linking the localities passing by the walls of the fortress. The first archaeological surveys in the Capidava area started at the beginning of the 20^th^ century and were initiated by Vasile Pârvan and Grigore Florescu^10^. Archaeological research in and near the Capidava fortress has started in 1924 and continues without major interruptions to date, but until recently the biological material discovered here was not fully exploited. The archaeological excavations have highlighted dramatic changes of the fortress structure since it was built at the end of the third century AD^10,11^. At the end of the 6^th^ century AD, after the Huns burned down the fortress, a small quadrilateral fort occupying the southern quarter of the precinct was built. A century later, amid the attacks of Avars and Slavs, the fortress was completely destroyed and abandoned by the last troops overseeing the area. In the 10^th^ century another wave of destruction and rebuilding was recorded, this time the walls being reconstructed following the initial pattern used in Roman times^11^. During the 10^th^-11^th^ centuries, the Lower Danube Basin was politically part of the Byzantine Empire. This was a troubled period, with complex interactions between several populations (Bulgarians, Kievans, and Turkics). As Capidava fortress is placed on the fringe of the Danubian Byzantine Empire, many migratory populations used this settlement in their passage^12^. Due to lack of solid archaeological evidence, particularly absence of any grave goods, the individuals from the C58 archaeological complex, the subject of this study, cannot be associated to any invasive ethnic groups or to any local populations (Bulgarians, Vlachs, Byzantines etc.).

## Results

### Archaeological context and osteological analysis

The assemblage of human skeletal remains belonging to the ten individuals was placed in a multiple burial pit (C58) and was not arranged according to the Christian burial customs of the medieval period, as observed in the majority of the graves from this archaeological site (simple, quadrilateral inhumation graves, generally west-east oriented skeletons in dorsal decubitus). The skeletons were found on the bottom of an elliptical pit, with no artifacts present (Supplementary Fig. S2). It is highly likely that the individuals were buried reasonably soon after death, as they were partially articulated, spatial position of body segments was kept after skeletonization, no animal predation marks were observed on bones, and no secondary disturbances were detected in the stratigraphy. The C58 archaeological complex is located near similar structures (circular pits) with potential uses as storage, waste disposal or loess exploitation (Supplementary Fig. S1). Radiocarbon dating results show that the analyzed funerary complexes were not contemporaneous, the C58 multiple burial pit being older (773–906 cal. AD) than the C143 single grave (962–1040 cal. AD). Previous radiocarbon dating is available for Cap-M4 (880–990 cal. AD) an individual buried according to Christian funerary custom in terrace B of the necropolis^7,12^.

The anthropological analysis revealed that the skeletal material from the C58 grave can be attributed to a minimum number of ten individuals. All results pertaining to the osteological analysis are summarized in the Supplementary Table S1. The skeletons were conserved in proportions ranging from 23 to 86%, and missing osteological inventory was absent exclusively due to taphonomic reasons. Case descriptions are only briefly outlined here because an osteological catalog for all individuals recovered since 2010 until present will be subsequently published together with a more thorough analysis of the archaeological contexts for this site. The skeletal material is composed of seven adult and three subadult individuals. There were six adults for which the sex could be estimated based on the cranial features and the morphology of the pelvic bones^13^. Sex distribution is rather imbalanced, as four of the individuals displayed male typical morphological features (Cap-C58-1, Cap-C58-2, Cap-C58-3, and Cap-C58-5) and the remaining two exhibited skeletal markers which indicate that they were probably females (Cap-C58-6, and Cap-C58-11). This small group of individuals is pretty diverse in terms of age since only the old adult category is absent. The male individuals recovered from the C58 funerary assemblage exhibited signs of violent traumas (Supplementary Fig. S3). The bioarchaeological analysis revealed the presence of two antemortem injuries on the scapula of two individuals, both with infection signs, and four perimortem wounds, one of which was identified on the posterior lateral epicondyle of C58-5s’ left humerus. Each of the C58-1, C58-2, and C58-5 male individuals displayed one perimortem trauma on the frontal bone, two of which were sharp force traumas (C58-1 and C58-5) and one blunt force trauma (C58-2).

### Samples and sequencing

We successfully reconstructed the complete mitochondrial genomes for all six individuals from the Capidava multiple burial selected for molecular analysis. The bioinformatics processing of the raw reads reveals that the resulted sequences for all analyzed samples reach standard quality requested to assure the authenticity of the NGS data. The details of NGS data for each sample are summarized in the Supplementary Table S2. Using hybridization capture in solution^14^ combined with high-throughput sequencing, six whole mitochondrial genomes were generated with the depth of average coverage ranging from 69x to 176x. The DNA damage profile of the samples and average fragment length were used as criteria of authentication in an iterative probabilistic approach^15^ that allows to estimate human contamination and to reconstruct the endogenous mitochondrial DNA (mtDNA) sequences. The rates of cytosine deamination at the 5′ end of the molecules were high, varying from 24.7% to 41.3%. The resulted mtDNA fragments are short, with a median length of 57.87 base pairs (bp) which ranges in the typical size length for aDNA^16^. In addition, the percentage of possible present-day human contamination was evaluated in order to further asses the authenticity of the ancient mitochondrial genomes. No significant levels of contamination were detected, as shown in the Supplementary Table S2.

The mutation patterns revealed by the analysis of the complete mitochondrial genomes of six samples from the C58 archaeological complex can be classified into five macro-haplogroups (H, J, N, T, and U) (Supplementary Table S3). The maternal genetic composition of the Capidava multiple burial appears to consist of six different west Eurasian haplogroups (H11a1, J1c15, N1a3a, T2b, U4d2, and U6a1a1) with no east Eurasian mitochondrial lineages detected.

### Phylogenetic analyses

A total number of 306 complete human mtDNA records (Supplementary Table S4) were found to match the maternal lineages of the investigated medieval individuals and were used for the construction of phylogenetic networks in order to outline the geographic affinities and genetic relationships of the Capidava multiple burial individuals. A small number of equivalent haplotypes from literature could not be properly aligned due to incomplete sequences, and these were not used in the phylogenetic networks.

MtDNA sequences of medieval Capidava multiple burial were depicted in six Median-Joining Networks. Samples falling within the same sub-haplogroup as those studied are emphasized by a blue circle. The networks are color-coded according to the “Traits block” of the Nexus-format alignments. Three different attributes (represented by three different colors) were used in order to discern between the samples analyzed in this study and other present-day or historical samples. Red nodes correspond to the Capidava samples, orange circles represent ancient samples and the modern ones are colored green. Black circles represent hypothetical haplotypes. The sizes of the circles are proportional to the frequency of each haplotype; the smallest colored circles representing only one sample. The numbers of cross lines in the branches between adjacent nodes denote number of mutations. The length of the branches is not relevant as they were adjusted to fit the page. The closest samples to those studied are labeled with GenBank accession number and their geographical origin.

**Figure 1:**
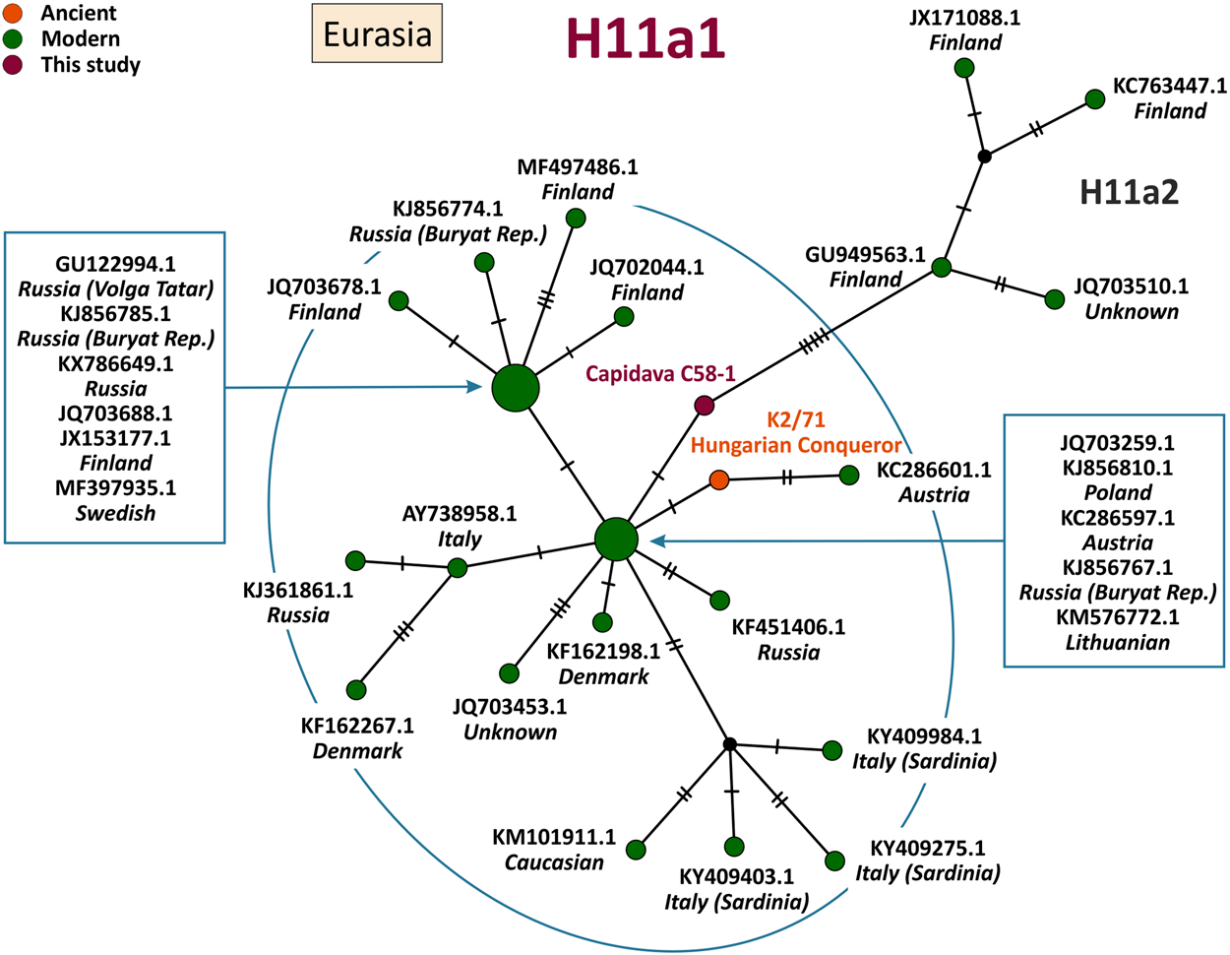
Median joining network for haplogroup H11a1 obtained from complete mtDNA genomes.

**Figure 2:**
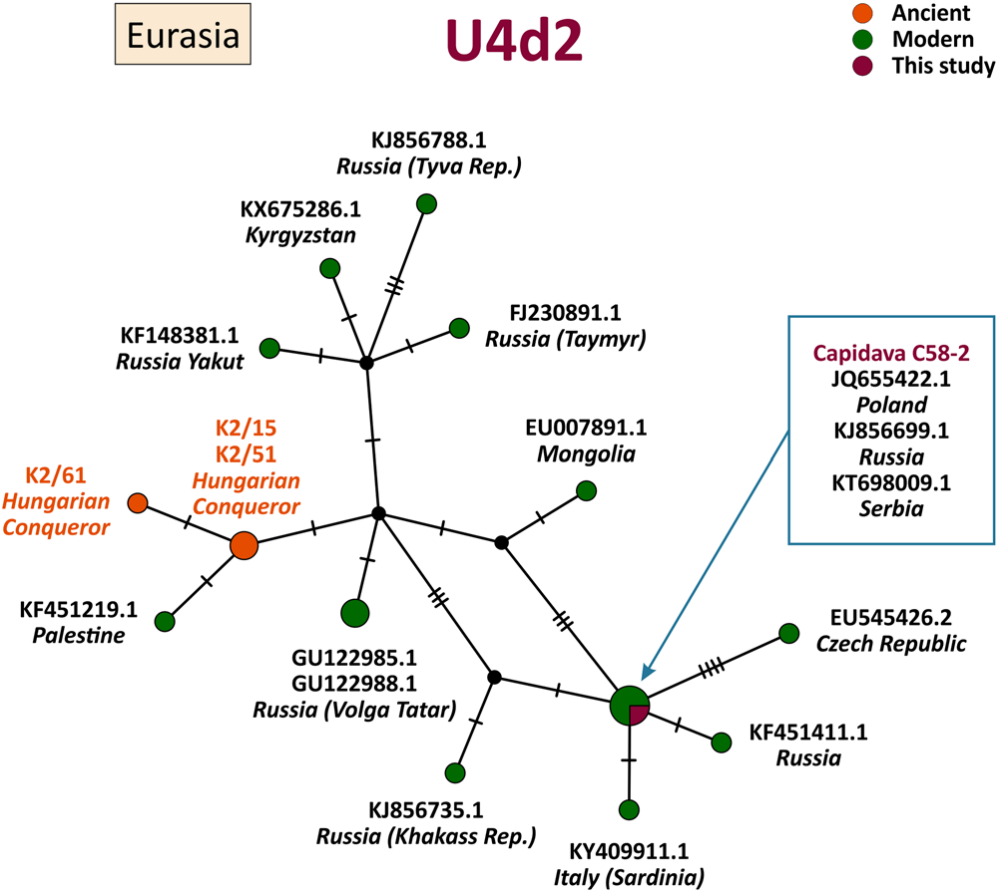
Median joining network for haplogroup U4d2 obtained from complete mtDNA genomes.

**Figure 3:**
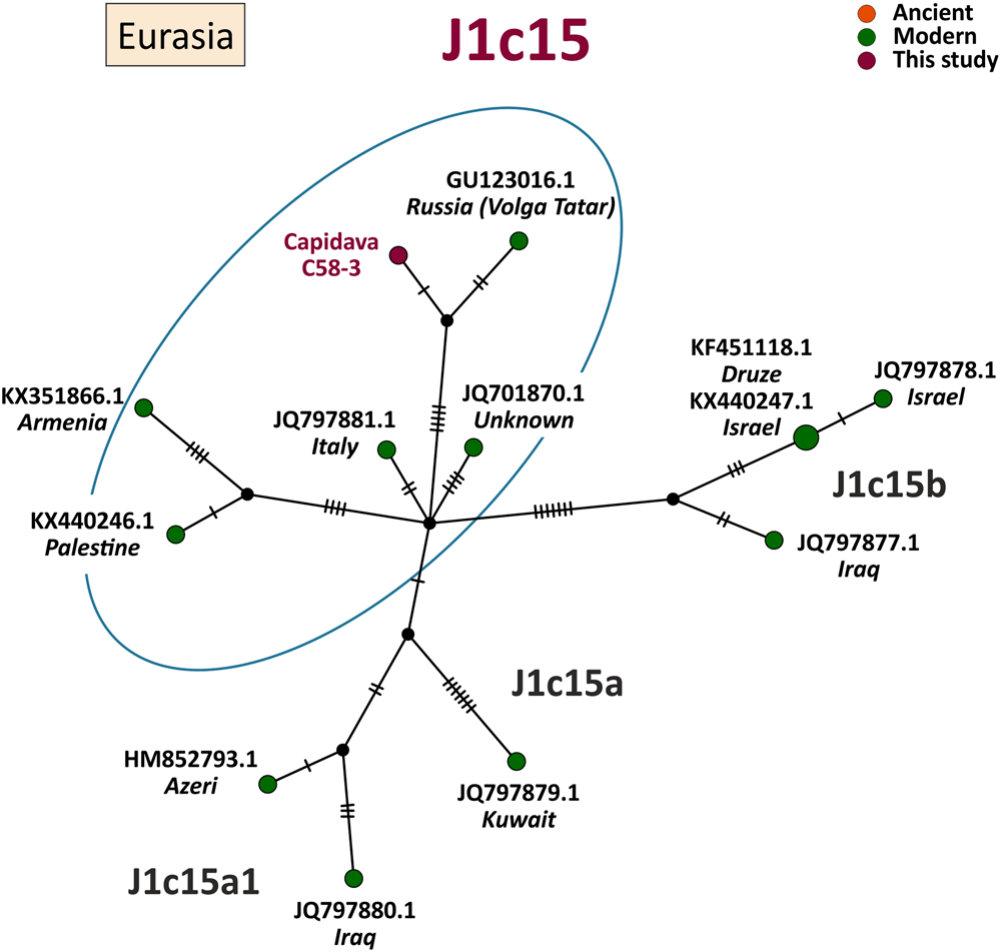
Median joining network for haplogroup J1c15 obtained from complete mtDNA genomes.

**Figure 4:**
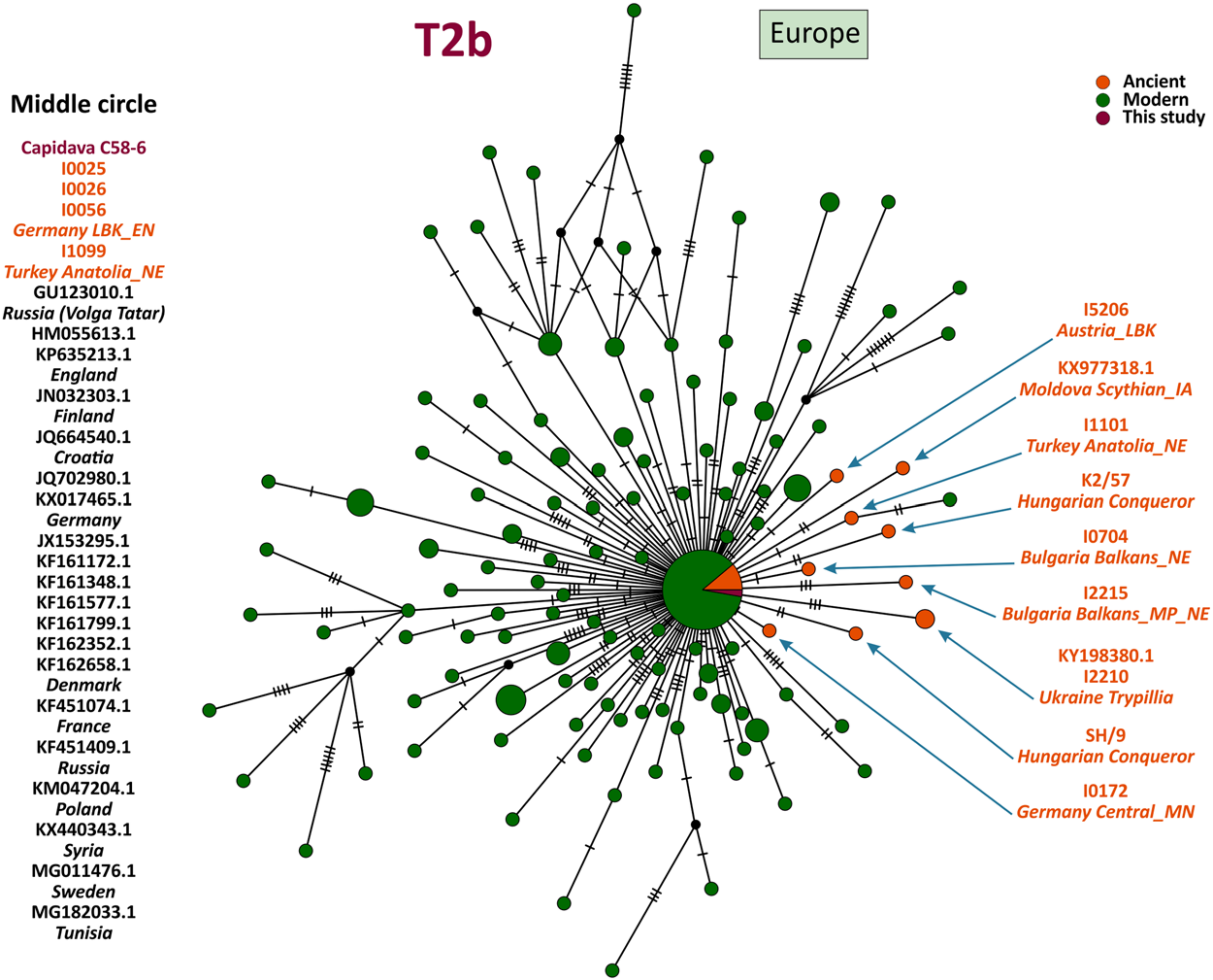
Median joining network for haplogroup T2b obtained from complete mtDNA genomes.

**Figure 5:**
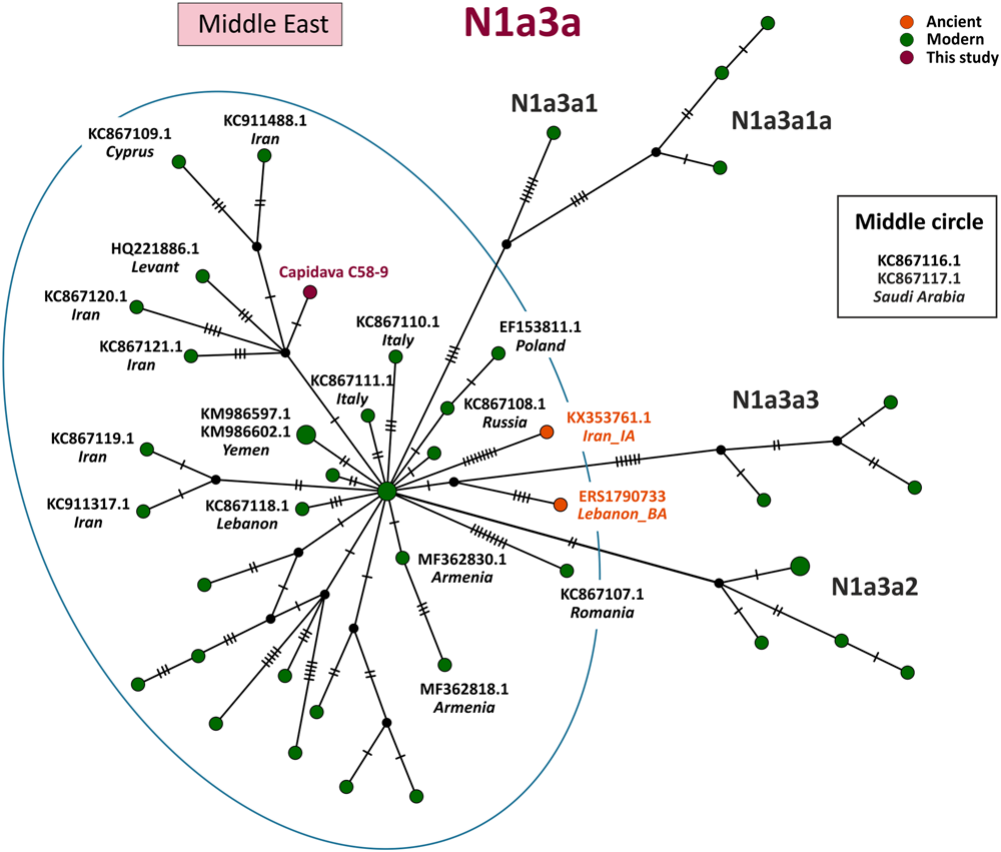
Median joining network for haplogroup N1a3a obtained from complete mtDNA genomes.

**Figure 6:**
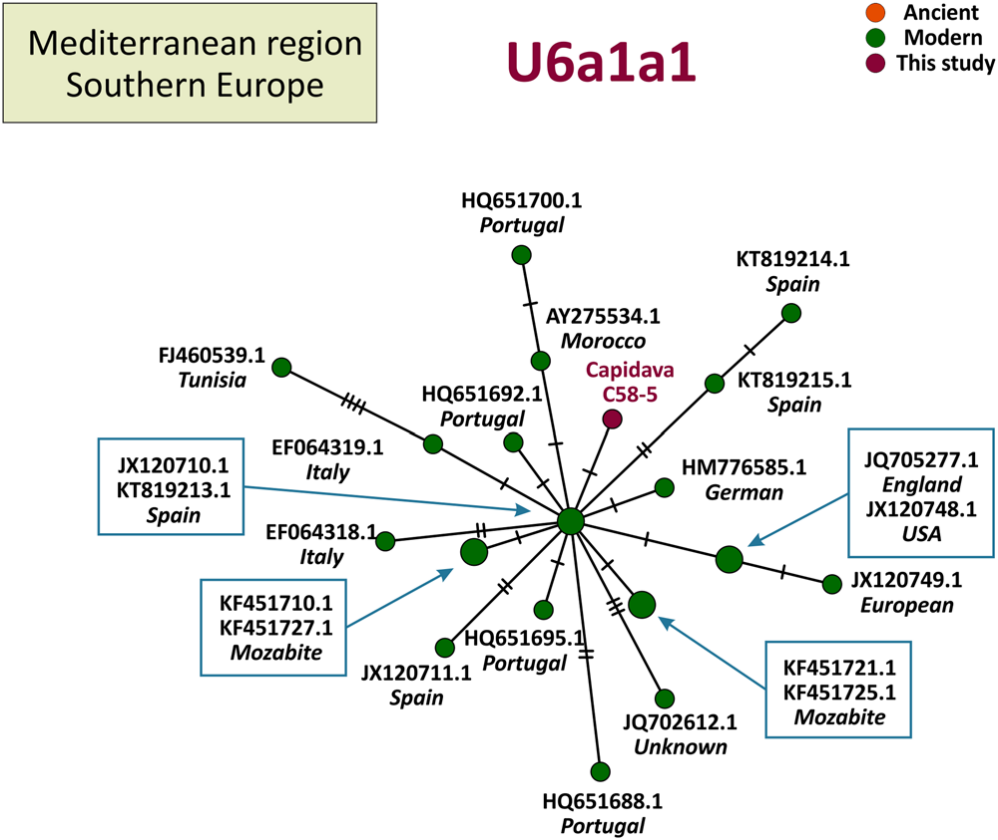
Median joining network for haplogroup U6a1a1 obtained from complete mtDNA genomes.

The increased level of resolution of full length mtDNA data allowed for the identification of direct links among closely related ancient and modern mitogenomes and for the precise assignment of geographic affinities. Phylogenetic analysis showed that three C58 haplogroups, H11a1 (Fig. 1), U4d2 (Fig. 2), and J1c15 (Fig. 3), are ubiquitous in Eurasia, while the T2b clade is widely spread across Europe (Fig. 4). The closest sequence matches elucidated using median-joining networks pointed to geographic affinities restricted to the Middle East in case of N1a3a (Fig. 5) and to the Mediterranean region for U6a1a1 (Fig. 6).

### Population genetics analyses

**Figure 7:**
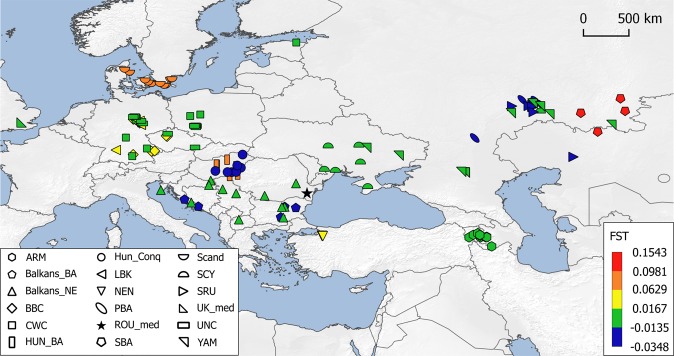
Heatmap of F_ST_ and geographic distribution. Smaller pairwise F_ST_ values indicating less genetic distances are marked by blue shades. Exact F_ST_ values and population information are listed in Supplementary Table S6. The map was created using QGIS 2.18.11 (QGIS Development Team, 2016. QGIS Geographic Information System. Open Source Geospatial Foundation. http://qgis.osgeo.org).

Pairwise genetic distances were calculated based on the complete mitochondrial genomes of 18 ancient Eurasian populations, including the medieval Capidava group (Supplementary Table S5). Due to the fact that our investigated population is small (six samples) we grouped it together with four of the formerly published medieval samples from this necropolis^7^. The medieval population from Capidava showed the lowest distances from four ancient populations: Bronze Age population of the Balkan Peninsula (Balkans_BA) (F_ST_ = −0.0348), Srubnaya (SRU) (F_ST_ = −0.02367), Poltavka-Potapovka (PBA) (F_ST_ = −0.01677), and Hungarian Conquerors (HUN_Conq) (F_ST_ = −0.01325); these values were not significant (p > 0.05). Five ancient groups (SBA, Scand, NEN, HUN_BA, and BBC) appeared most distant to the individuals in our dataset, displaying significant differentiation (p < 0.05) (Fig. 7). The exact F_ST_ values and their corresponding p-values are listed in the Supplementary Table S6.

**Figure 8:**
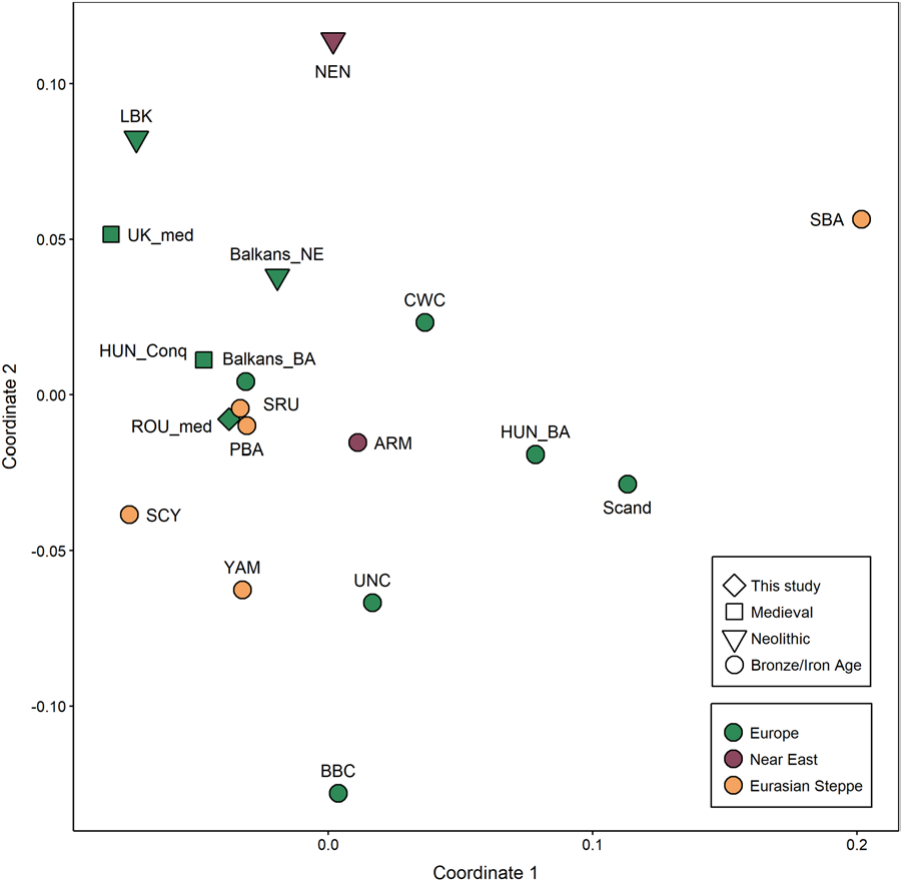
MDS plot based on linearized Slatkin FST values calculated from complete mtDNA genomes. Stress value is 0.142. Detailed description of each individual from each population used in comparison is given in the Supplementary Table S5, while the linearized Slatkin FST values are listed in the Supplementary Table S6.

These genetic distances were visualized by displaying the linearized Slatkin F_ST_ values on a multi-dimensional scaling (MDS) plot (Fig. 8). The resulted plot reflects the extremely tight connection between the ROU-med and Bronze Age population from the Pontic-Caspian steppe (Late Bronze Age Srubnaya culture and its ancestors Potapovka and Poltavka). The investigated medieval dataset also shows strong affinities to the Bronze Age population from the Balkans and to the Hungarian Conquerors, groups which are geographically close to the medieval population from Romania. Near Eastern Neolithic, Sintashta-Andronovo, and Bell Beaker seem to be the most distant populations relative to the Capidava group.

## Discussion

The analysis and interpretation of the available bioarchaeological data provides some evidence regarding past human interaction patterns and mortuary practices, while genetic analysis points out biological relationships among individuals. However, it is only possible to speculate about the reasons for which the C58 individuals, who died at roughly the same time, were buried together in such a special and rare funerary context. They showed no clear pattern of organization which suggests that they were rather disposed of into the pit, as opposed to ritual deposition, possible due to haste and/or lack of consideration for the dead. In contrast, previously published medieval Capidava samples^12^, and yet unpublished samples were individually buried according to Christian rituals with few associated bronze artifacts. The pathological conditions identified on these human remains (dental enamel hypoplasia, *cribra orbitalia*, porotic hyperostosis, dental disease, periosteal inflammation, and osteolytic lesions on the vertebrae) possibly reflect the poor life quality of this group of individuals. When comparing the presence of pathological features between the individuals from the C58 complex and the ones from the rest of the necropolis, a slight increase in the incidence of stress indicators can be seen. *Cribra orbitalia* has an incidence of 20% in the C58 group and 17% in the necropolis group, while porotic hyperostosis occurs in 50% of the individuals from the C58 complex and in only 7% of the individuals from the necropolis. Linear enamel hypoplasia is present in 18% of the individuals from the necropolis, while in the C58 complex, it occurs in 10% of the cases. It is very likely that they had experienced one or more of the following: nutritional deficiencies, physiological stress during childhood, repetitive physical activity, and low immunological status. With regard to trauma and fractures, 8% of the individuals from the necropolis group suffered cranial trauma, while a much higher incidence, 30%, can be seen in the C58 complex. All adults show mild signs of osteoarthritis, mainly on the long bones of the upper and lower limbs, which can be associated with moderate physical activity during their lifetime. The marginal social status (*e*.*g*. outsiders, heretics, prisoners, slaves, poor, etc.^17^) of this group at the time and place of their deaths seems a reasonable explanation when correlating the inconsiderate burial with the observed pathologies. The pattern of physical trauma observed here indicates a violent death for nearly half of the individuals, all of them males, which might be the result of some kind of skirmish. A higher frequency of skeletal trauma in males in comparison to females is not unexpected, because they were usually involved in more physically challenging and dangerous activities than females^18^. The prevalence of frontal craniofacial injuries determined in all analyzed male individuals could be a consequence of an imbalanced fatal physical confrontation. This injury pattern indicates that these individuals were not attacked from behind (execution style). Cuts on the humerus and rib of Cap-C58-5 suggest he might have been involved in a physical struggle. Skeletal evidence of deliberate interpersonal violence was previously recorded, being linked to different historical periods such as Neolithic multiple and collective graves from Germany^19^, a Copper Age mass grave from the territory of Croatia^20^, and Early Iron Age settlements from Dobruja^17^. The number of similar cases that date to the medieval period is limited to few notable examples^21^. For instance, the first attempt at identifying the reasons for the violent death of late medieval individuals buried in a mass grave from the University Square (Bucharest, Romania) was recently documented^22^. Comparable examples to the analyzed sample set in terms of demographic profiles (high number of women and children) were best explained by raids^23^, attacks that usually result in killing everyone encountered^24^. The disorganized body positions and the absence of religious rites specific to the medieval period observed in the C58 complex suggest that these individuals were perhaps outsiders (geographical, social or religious), unwanted or without rights in the local community whose representatives are not evident, at this point, in the adjacent necropolis.

A very similar multiple burial was discovered in the proximity of Capidava fortress in the 1959 archaeological campaign by Florescu *et al*. and published three years later^25^. The exact location of the multiple burial and the spatial relationship to the C58 burial cannot currently be accurately identified based on the published landmarks. This grave contains, according to the authors, a minimum number of nine individuals, similarly disposed of as the remains in C58, 8 adults and 1 child with possible supplementary remains belonging to one or more infants. Analyses concerning sex, age at death and traumas of these individuals were not recorded in this or ulterior publications. Nevertheless, a small paragraph pertaining to the position of the skeletal remains of a child from this grave (1959) raises the hypothesis that it may have been decapitated. In contrast to the multiple burial discussed in the present paper, the 1959 burial contained also fragments of metallic armour used in the archaeological dating of the context at 9^th^-10^th^ century AD^25^. The 1959 multiple burial human remains were not available for genetic analyses. The presence of two similar archaeological contexts dated to approximately the same age, by different means, seems to hint to a period of local turmoil and vulnerability for local and/or transiting groups.

The nucleotide substitution patterns displayed by the complete mitochondrial genome of each C58 individual, the absence of identical haplotypes amongst them, show the lack of close maternal relationships between the studied samples. Other kinds of biological relationships (*e*.*g*. paternal) cannot be excluded. Four individuals (three subadults and one female) were not selected for aDNA analysis because of their poor preservation and absence of teeth.

None of the haplotypes present in the multiple burial individuals matches the other five whole mitochondrial genomes found in the previously published samples^7^ (Supplementary Table S5) or the haplogroups that were identified by the analysis of the control region sequences in the other five individuals previously published^7^. These two groups of individuals which have been discovered in the same site, but in different archaeological contexts (C58 Information Center point and individuals from B terrace) share two macro-haplogroups, namely N and U. The higher level of classification based on complete mtDNA data revealed that the derivative lineages clustered under N macro-haplogroups are distinct (N1a3a: Cap-C58-9; N9a9: Cap-M9 and Cap-M11) and have different phylogenetic backgrounds. Two lineages descending from the common ancestor U were found in each set of individuals (U4d2 and U6a1a1 were detected in the multiple burial samples, while U5a1c2a and U3a were identified in the previously published samples^7^). Derivative sub-branches of haplogroups J and T which are commonly distributed across Europe were exclusively found in the multiple burial individuals of all genetically processed samples from this necropolis. Haplogroup H, the most widely spread and diverse maternal lineage in Europe was observed in lower frequencies among the samples from C58 complex than in the other medieval samples from Capidava. The influence of the maternal familial relationships on the funerary ritual is suggested by the presence of identical haplotypes only in adjacently buried individuals (single graves)^7^.

All the haplogroups identified in the ancient samples from this study are also present in the modern Romanian population as shown by the mtDNA control region sequences of 714 Romanians^8^. The only matching haplotype is T2b which was detected in modern samples from all historical provinces of Romania, except Dobruja. Most probably the other haplotypes could not be detected in modern samples due to the small resolution of the control region data and/or to low frequencies of these haplotypes in the modern population. Of all haplogroups described in this study, the most frequent one in the modern Romanian population is J1c, which is most common in present-day Dobruja individuals. Haplogroups H11 and U4 are more frequent in modern Walachia and Moldavia than in Transylvania, while N1a is absent from the Transylvanian set.

The Cap-C58-1 sample contains all defining mutations for H11a1, a descendant subclade of the common western Eurasian H11 haplogroup, as well as a private mutation 16261 T. Similar genetic profiles are currently distributed across most of the northern, central and eastern Europe and in Central Asia. H11a was identified in a Mesolithic hunter-gatherer of the Narva culture from Lithuania (Spiginas1)^26^, in a Bronze Age sample from Hungary (Rise247)^2^ and was also found associated to the Unetice culture (BZH1)^27^. H11a1 was found in a medieval sample belonging to an adult female of the Hungarian Conquest period^9^, but it is not an exact match to the C58-1 sample (Fig. 1).

The U4d2 haplotype is currently distributed in some geographic areas of Eurasia, being most frequent in populations of Eastern Europe (Volga Tatars and Slavic speaking groups) and in aboriginal Siberians (Yakuts, Tuvinians, Khakassians) (Supplementary Table S4). It has been suggested that U4d2 branch might be of Slavic origin due to its high incidence in Slavic-speaking populations and it might have expanded into Central Europe and Siberia from Eastern Europe^28^. This haplotype includes three ancient maternally inherited genomes, all from Hungarian Conquerors^9^. The haplotype of Cap-C58-2 contains all of the expected U4d2 diagnostic mutations aside from 16189 C, and four personal transitions. The same maternal genetic signature occurs in present-day Slavic populations (Poland, Russia, and Serbia) (Fig. 2).

The polymorphism distinguished in the complete mtDNA sequence of Cap-C58-3 can be classified as J1c15, a derivative sub-branch of the most prevalent form (J1c) of J macro-haplogroup in present-day Europe. This specific haplotype variant is rare in modern mtDNA data and seems to be connected mainly to the Near East, more sparsely to Europe and, to our knowledge, is absent from the published ancient datasets. The detected J1c15 haplotype is most similar to a sample observed in the gene pool of Tatars inhabiting the territory of the middle Volga River^29^ (Fig. 3).

Mitochondrial consensus sequence of Cap-C58-6 belongs to the T2b haplogroup, a major sublineage of T2 that spread into Europe from the Near Eastern refuges^30^. Equivalent haplotypes were found in remains from the Linear Pottery culture (LBK) in Central Europe^31^ and in an Early Neolithic skeleton from north-western Anatolia (I1099)^1^ (Fig. 4). The same T2b haplotype was identified in various present-day European populations, which could indicate its European origin.

N1a3a sub-branch defined by the 16201 T substitution was detected in the Cap-C58-9 sample. The phylogenetic network analysis (Fig. 5) reveals that the Capidava sample which lacks only the 207 A defining mutation for N1a3 is most closely related to modern samples originating from Iran, Levant, and Cyprus. The current geographic distribution of this maternal lineage shows that it is mainly restricted to the Middle East, being extremely rare in central-east Europe (Romanians, Poles, Belarusians, and Tatars of the Volga-Ural region), being indicative thus of Middle Eastern genetic traces in Europeans^32^. Ancient N1a3a haplotypes were documented in an Iron Age sample from Iran^33^ and a Middle Bronze Age individual from Lebanon^34^.

Cap-C58-5 displays the mutational motif for U6a1a1, a derived lineage nested within U6a1 which is spread from the Near East to northwestern Africa^35^. This subclade (U6a1a1) currently occurs at high frequencies in the central-western Mediterranean range as shown in Fig. 6. Its highest frequencies across the European continent are restricted to the Iberian and Italian peninsulas. This particular mitochondrial variant was not found in the published ancient mitogenomes, but other U6a haplotypes were previously reported for six Taforalt individuals attesting their preHolocene presence in the Maghreb^36^ and basal haplogroup U6 has been identified in a 35,000-year-old *Homo sapiens* from Romania (Muierii cave)^37^.

The maternal genetic composition of the Capidava population as represented by all analyzed samples points to a mixed origin from multiple sources. The high-resolution mtDNA data of the individuals buried in single graves in the necropolis, display genetic affinities towards Central and Eastern Europe and to Central Asia^7^. In contrast, according to the phylogenetic analysis of the C58 haplotypes, multiple connections with ancient Middle East mtDNA genomes from different historical periods were found^1,33,34^, as well as links to the medieval Hungarian conquerors^9^. Close genetic ties to the Conquerors were also exposed by the population genetic analysis (Figs 7 and 8). It is worth noting that the medieval Hungarian samples used for genetic distances are different from those used for phylogenetic reconstruction, a fact which increases the strength of this apparent connection. It has been argued by Neparáczki *et al*.^9^ that the best fitting origin of the European component identified in Hungarian Conquerors are the Late Bronze Age Srubnaya culture, and its ancestors Potapovka and Poltavka. Very tight relationships were observed between the medieval Capidava population and these Bronze Age populations of steppe origin. These data suggest that the Pontic-Caspian steppe genetic input in Eastern Europe might not be solely restricted to the Carpathian Basin.

## Conclusion

This study provides new anthropological and genetic insights on a medieval group of individuals from southeastern Europe, collectively buried in a particular fashion that contrasts the typical Christian funerary rituals of this historical period as observed in the same necropolis. The unusual position of the human skeletal remains, associated pathologies, and the presence of traumatic injuries among the male individuals, point to interpersonal violence, poor life quality, and the careless treatment of the dead. The high diversity of mitochondrial haplogroups detected in the C58 archaeological complex (six individuals, six haplotypes) reflects the lack of close maternal relationships among individuals, and quite an intricate genetic history, as the identified subclades have diverse geographic distribution (Eurasia, Middle East, Southern Europe). The full length mitogenomic data reconstructed in this study enriches the available genetic information for the medieval period, particularly for Dobruja. All the existing mtDNA information on the medieval Capidava population provides further evidence for genetic connections to the Hungarian Conquerors, a Bronze Age population from the Balkans and Bronze Age populations of steppe origin (Srubnaya and Potapovka-Poltavka).

## Methods

### Archaeological background and samples information

The collection of archaeological human remains analyzed and reported in this study was unearthed from the medieval Capidava necropolis (sector X, *extra muros*, Information Center point, C58 archaeological complex) (Supplementary Fig. S1), located in the Dobruja region, Constanța County, Capidava village, during the preventive archaeological research performed in 2015 as part of the preservation and restoration project of the Capidava fortress. The archaeological survey was authorized by the Romanian Ministry of Culture and National Identity through the National Commission of Archaeology (permit number 62/30.03.2015/M.C.). A detailed preliminary archaeological report is available in Romanian at http://cronica.cimec.ro/ (*Cronica Cercetărilor Arheologice Din România* 2016, edited by the National Institute of Heritage). All human remains belonging to ten individuals discussed in this paper have been transferred to a permanent collection deposited for research at the Molecular Biology Center, Interdisciplinary Research Institute on Bio-Nano Sciences, “Babeș-Bolyai” University in Cluj-Napoca and carry the following codes: Cap-C58-1, Cap-C58-2, Cap-C58-3, Cap-C58-5, Cap-C58-6, Cap-C58-7, Cap-C58-9, Cap-C58-10, Cap-C58-11. The collection is open to researchers, upon request.

A total number of 186 funerary units were discovered during the above mentioned archaeological excavations (sector X, *extra muros*, Information Center, Access way and Parking lot points), most of them being simple, quadrilateral inhumation graves that contain a single individual. With the exception of two Bronze Age individuals, the rest of them were dated to the medieval period based on funerary rituals, grave goods or subsequent radiocarbon dating. Peculiar for this necropolis is the presence of a circular multiple burial pit containing the skeletal remains of ten individuals which are the subject of this study (Supplementary Fig. S2). A direct radiocarbon dating was performed at Beta Analytic (Florida, USA), using the human osteological material retrieved from two graves, one containing a single individual showing signs of similar disregard towards the deceased (ventral decubitus), located in the proximity of the multiple burial (Cap-C143, Beta - 475779), and one from the multiple burial (Cap-C58-1, Beta – 475778). Radiocarbon dating for a Capidava individual buried in a typical single grave (Cap-M4, B terrace) was previously published^7^ and mentioned in the Results section. Details regarding the archaeological background and associated images can be found in the Supplementary Information.

### Osteological analysis

All bone fragments and dental samples recovered from the Capidava multiple burial were morphologically evaluated at the Interdisciplinary Research Institute on Bio-Nano Sciences, “Babeș-Bolyai” University in Cluj-Napoca. The anthropological investigation was carried out at individual level in order to gather information on age at death, sex, traces of injuries, pathological conditions, and stress markers that can infer the demographic structure and epidemiological conditions of this group of medieval individuals. The osteological examination was performed using previously described standard guidelines^13,38^ and the results are summarized in the Supplementary Table S1. The availability of dental specimens with good preservation status was an eliminatory sample selection criterion for ulterior molecular genetic investigations. Therefore, aDNA analysis was not attempted in case of four archaeological individuals (Cap C58-4, Cap C58-7, Cap-C58-10, and Cap-C58-11).

### DNA extraction and sequencing

Six specimens consisting of intact multi-rooted teeth (labeled as Cap-C58-1, Cap-C58-2, Cap-C58-3, Cap-C58-5, Cap-C58-6, and Cap-C58-9) were sampled and stored for genetic analysis during the archaeological excavation. All procedures leading to Next Generation Sequencing (NGS) were performed in dedicated aDNA facilities of the Molecular Anthropology Laboratory, University of Florence. Standard criteria for the analysis of aDNA^39^ were rigorously followed and strict precautionary measures were undertaken to avoid contamination with exogenous DNA. All experimental steps were implemented following the protocol described in detail by Modi *et al*.^40^ and Tassi *et al*.^41^. In brief, each tooth sample was cleaned by mechanical abrasion of the surface using a dental micro-drill, followed by UV irradiation (254 nm) for 45 minutes, on each side. The powdered tissue used for the isolation of genetic material was obtained from the tooth roots using the same dental device at a very low rotation speed. For each sample, DNA was extracted from 50 mg of biological material following a silica-based protocol, specially designed to target ultra-short molecules^42^. Illumina libraries were constructed using a previously developed protocol^43^. No enzymatic treatment for damage repair was applied in order to preserve and consequently analyze the degradation patterns of DNA fragments. The libraries were amplified to reach plateau and enriched for human mitochondrial DNA using a selective bead-capture approach by hybridization of aDNA with probes consisting of long-range PCR products^14^. A negative control was processed during all analytical phases to monitor for possible contamination. The enriched libraries were pooled in equimolar ratios with libraries from other samples and paired-end sequenced (2 × 75 + 8 + 8 cycles) using the Illumina MiSeq platform.

### Bioinformatic NGS data processing and authentication

To reconstruct the mitochondrial sequences we used *schmutzi*, an iterative approach that jointly estimates present-day human contamination and assembles the endogenous mitochondrial genome^15^. Forward and reverse reads were merged into single sequences with a minimum overlap of 10 bp between paired-end reads and the adapters were trimmed using SeqPrep^44^. Unmerged sequences and sequences shorter than 30 bp were discarded. BWA^45^ software package was used for mapping the filtered reads against the revised Cambridge Reference Sequence (rCRS, NC_012920.1^46^), applying recommended parameter settings for aDNA molecules “–l 1000 –n 0.01 –o 2”. After removing PCR duplicates, only sequences with a map quality score ≥30 were retained and used to align at unique positions along the reference sequence. Consensus sequences for the mitochondrial genomes of all samples were called using *schmutzi* (parameters: “–logindel 1–uselength”). DNA damage patterns at the ends of the molecules and average fragment length were taken into account to identify and call endogenous bases. Present-day human contamination estimates were performed using a non-redundant database of 197 human mitochondrial genomes available in the software package. Misincorporation patterns at the 5′ and 3′ ends were computed using contDeam, a program provided with *schmutzi* packages. A summary of NGS data for each analyzed sample can be found in the Supplementary Table S2. Mitochondrial haplotypes for each sample were determined with the use of HaploGrep^47^, based on PhyloTree^48^ build 17 (Supplementary Table S3). Consensus sequences corresponding to each medieval individual from Capidava were submitted to NCBI GenBank under the accession numbers MH298069-MH298074.

### Phylogenetic and population genetics analysis

The phylogenetic relationships of the six newly characterized mitogenomes and other modern and ancient published data were inferred from haplotype networks. For this purpose, we have searched in the available online databases and literature for sequences that correspond to the haplogroups identified in the Capidava multiple burial pit. All matching complete mtDNA sequences were downloaded from web depositories (NCBI Nucleotide, European Nucleotide Archive) or were requested from the authors (Supplementary Table S4). The haplogroup of all sequences was re-checked using HaploFind^49^ and we grouped them according to their maternal lineage. All nucleotide sequence subsets were aligned with MAFFT version 7^50^ applying the progressive method G-INS-1. Multiple sequence alignments were converted into Nexus format files with MEGA^51^ which were used to build median-joining networks in PopART^52^ with default settings. The phylogeographic relations were inferred based on the geographic origin of the most similar sequences from literature.

Another database of full-length mitochondrial sequences comprising 282 ancient samples from 17 relevant Eurasian populations was assembled from published literature (Supplementary Table S5) in order to evaluate the genetic similarities between them and the samples analyzed in this study. The samples were grouped according to their geographic and chronological affiliations. In cases where populations consisted of very low sample numbers, related neighboring groups were brought together, if possible. For this reason, the six Capidava samples analyzed in this study were clustered with previously described NGS medieval samples from the same archaeological site^7^ under the “Rou-med” umbrella. Pairwise population differentiation values (F_ST_) were calculated from complete mtDNA genomes using the Arlequin 3.5.2.2 software^53^. Nucleotide positions showing point indels (309.1 C(C), 315.1 C, AC indels at 515–522, A16182c, A16183c, and 16193.1 C(C)) were excluded from the analysis as they can cause difficulties when multiple mtDNA genomes are aligned. The most appropriate nucleotide substitution model and the estimate of gamma parameter were determined with jModeltest 2.1.10.^54,55^. The F_ST_ values of the ancient dataset were computed assuming a Tamura & Nei^56^ substitution model, 10000 permutations for p-value calculation and an allowed level of missing data of 0.05. The matrix of Slatkin F_ST_ values (Supplementary Table S6) was displayed on a multi-dimensional scaling (MDS) plot represented in a two-dimensional space (Fig. 8) using metaMDS function based on Euclidean distances implemented in the vegan library^57^ of R 3.3.2^58^. The F_ST_ values were also represented by different color shadings to indicate the degree of similarity or dissimilarity of the ROU-med population to the ancient Eurasian populations and visualized on a geographic map (Fig. 7).

## Supplementary information

Supplementary Information

Supplementary Dataset 1

## Data Availability

The dataset generated during the current study is available in the GenBank repository, under the accession numbers MH298069-MH298074.
