# Supplementary material for: Mitochondrial ancestry of medieval individuals carelessly interred in a multiple burial from southeastern Romania
Source: Sci Rep. 2019 Jan 30;9:961. doi: 10.1038/s41598-018-37760-8 (PMC6353917; doi:10.1038/s41598-018-37760-8)
Supplement: Supplementary file 1 — Supplementary Information [file 41598_2018_37760_MOESM1_ESM.pdf]

Mitochondrial ancestry of medieval individuals carelessly interred in a multiple burial from southeastern Romania

Supplementary Information

Ioana Rusu[1,2,*], Alessandra Modi[3,*], Claudia Radu[1,4], Cristina Mircea[1,2], Adriana Vulpoi[5], Cătălin Dobrinescu[6], Vitalie Bodolică[6], Tiberiu Potârniche[6], Octavian Popescu[1,2,7], David Caramelli[3], Beatrice Kelemen[1,2]

[1] Molecular Biology Center, Interdisciplinary Research Institute on Bio-Nano-Sciences, Babeș-Bolyai University, 400271 Cluj-Napoca, Romania
[2] Department of Molecular Biology and Biotechnology, Faculty of Biology and Geology, Babeș-Bolyai University, 400006 Cluj-Napoca, Romania
[3] Dipartimento di Biologia, Università di Firenze, 50122 Florence, Italy
[4] Department of Ancient History and Archaeology, Faculty of History and Philosophy, Babeș-Bolyai University, 400084 Cluj-Napoca, Romania
[5] Nanostructured Materials and Bio-Nano-Interfaces Center, Interdisciplinary Research Institute on Bio-Nano-Sciences, Babeș-Bolyai University, 400271 Cluj-Napoca, Romania
[6] Department of Research-Development and Projects, Museum of National History and Archeology, 900745 Constanța, Romania
[7] Institute of Biology Bucharest, Romanian Academy, 060031 Bucharest, Romania

* Correspondence and requests for materials should be addressed to I.R. (rusu.n.ioana@gmail.com) or A.M. (alessandra.modi@unifi.it)

SUPPLEMENTARY FIGURES

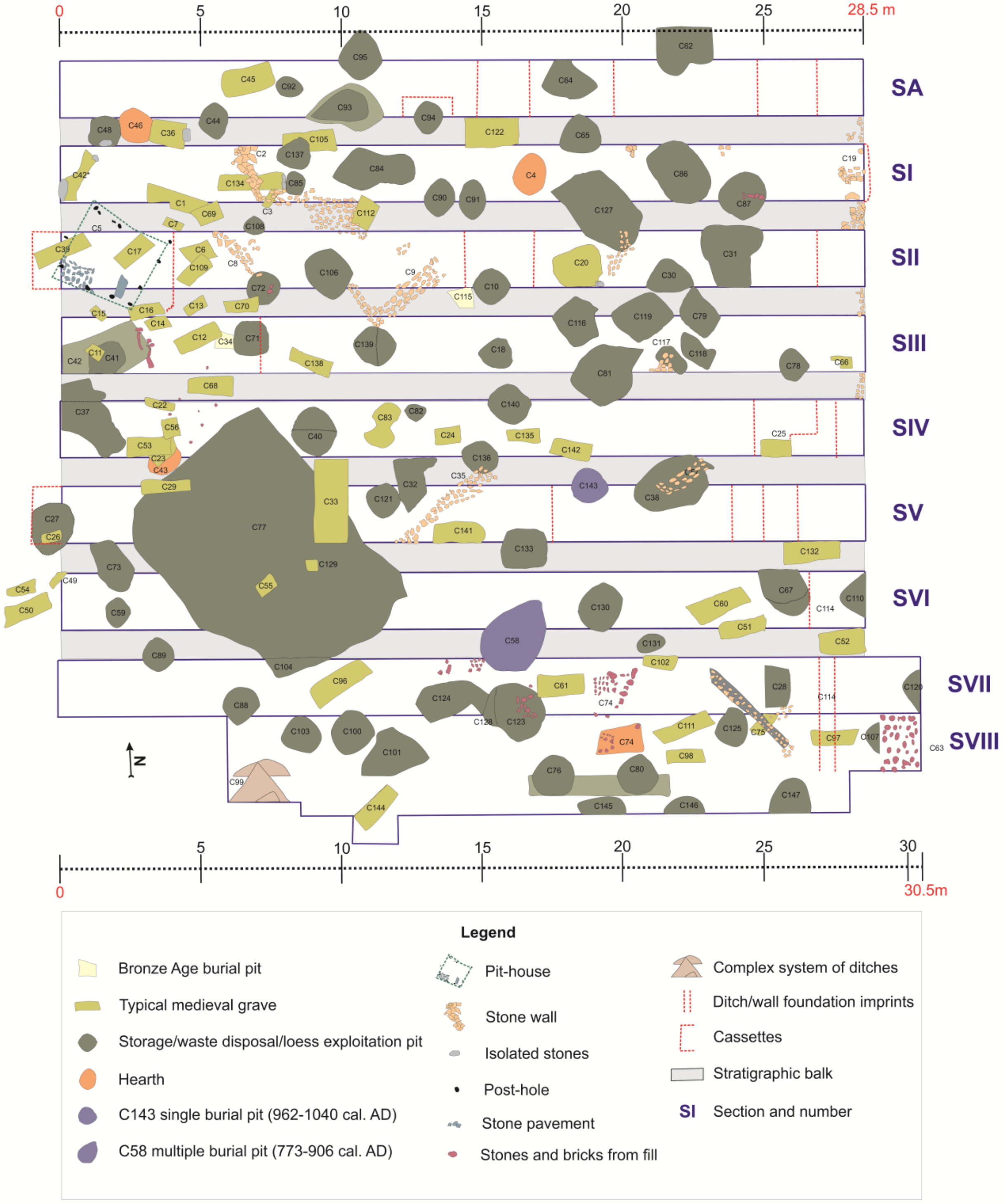

Supplementary Figure S1. Distribution of archaeological complexes within the Information Center area of the Capidava necropolis.

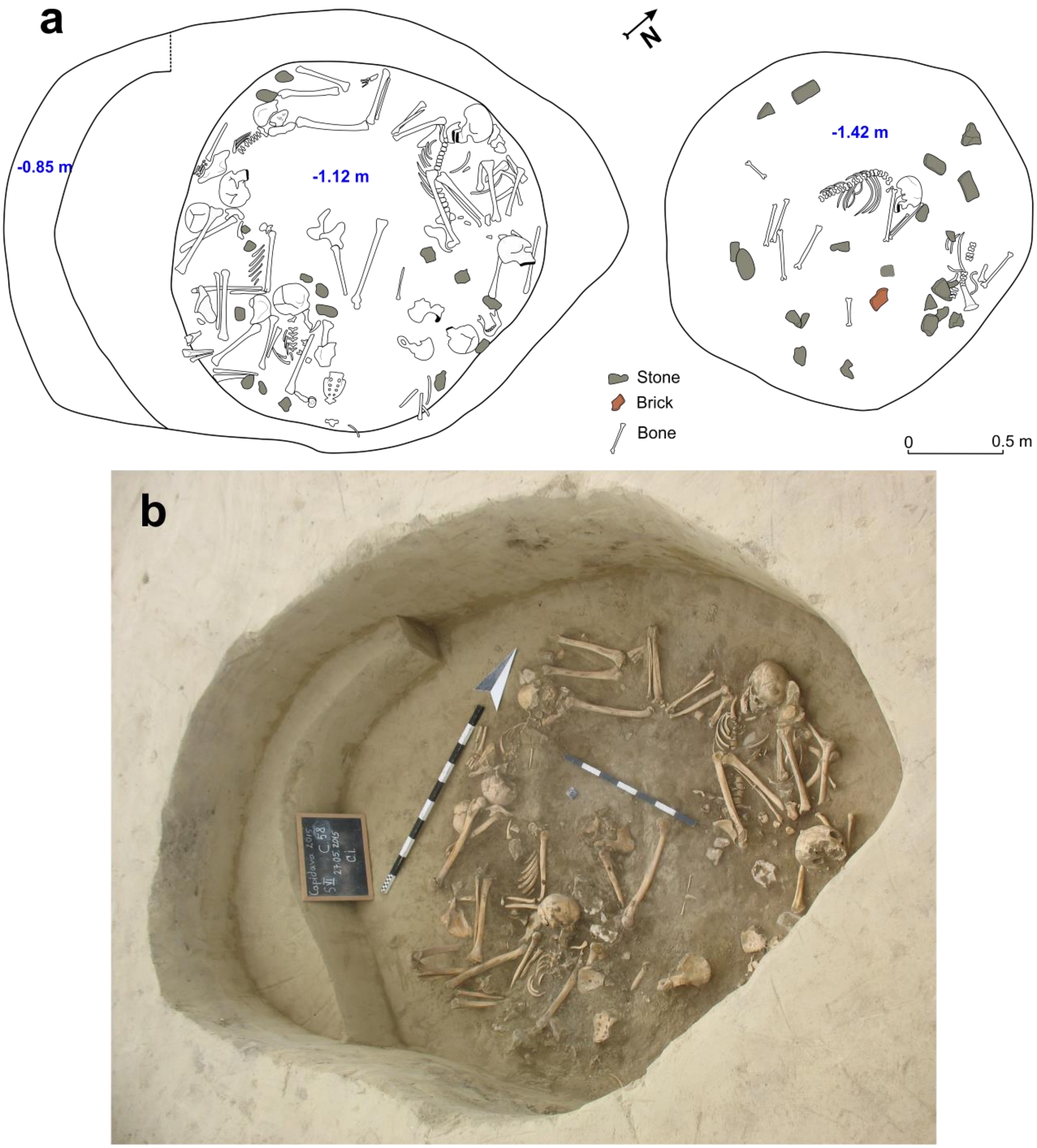

Supplementary Figure S2. Human remains of a minimum of ten individuals found in the C58 archaeological complex (Capidava necropolis, Information Center area). (a) Layout of the first (left) and second (right) levels of the multiple burial and (b) Photograph of the first level (-1,12 m) of the multiple burial.

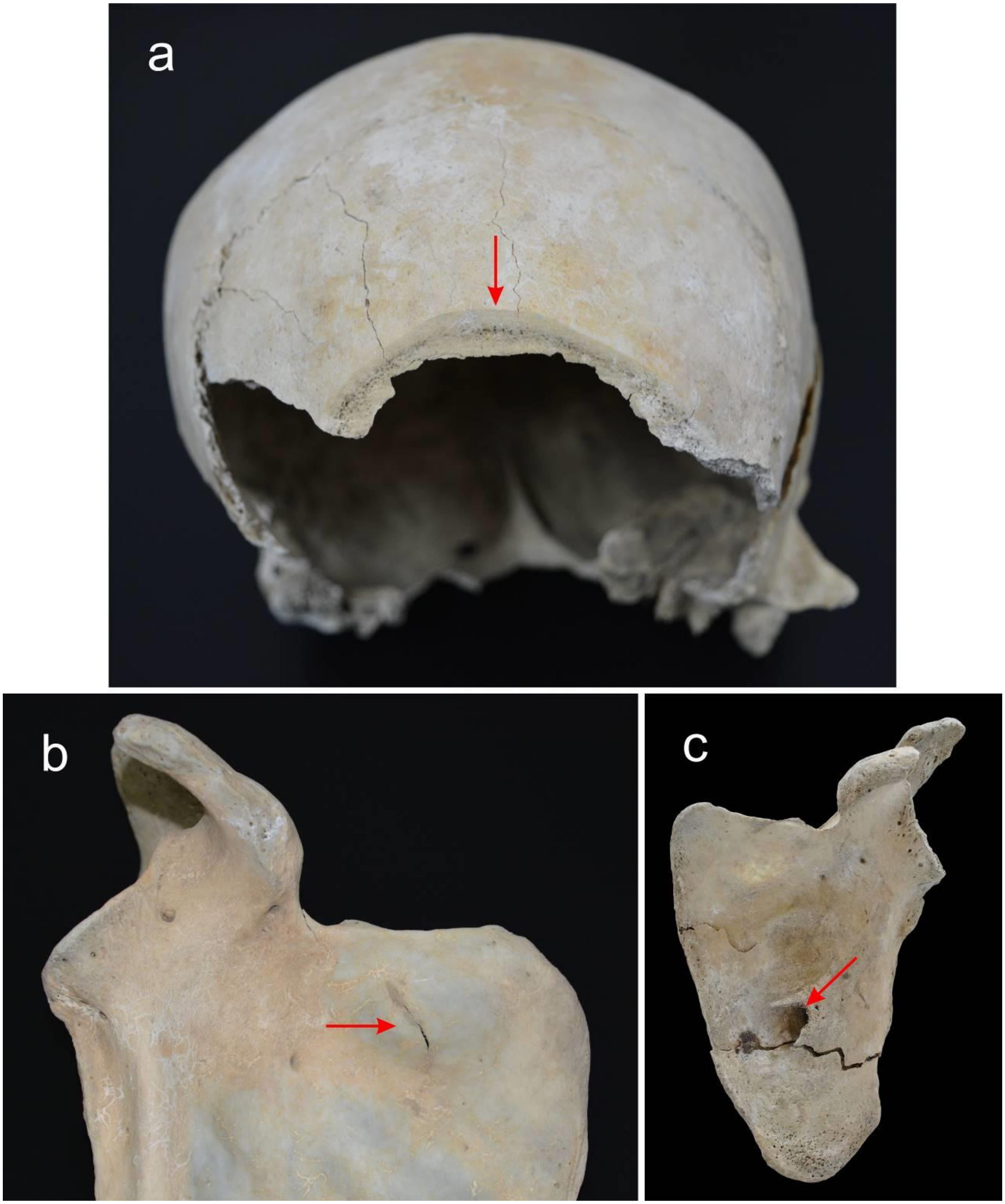

Supplementary Figure S3. Representative skeletal trauma of the analyzed samples (marked in red arrows). (a) Perimortem sharp force trauma at frontal bone, Cap-C58-1. (b) Antemortem cut on the right scapula, Cap-C58-1. (c) Antemortem penetrating trauma to the left scapula with infection signs, Cap-C58-3.
